# Laxative effects of fermented rice extract in rats with loperamide-induced constipation

**DOI:** 10.3892/etm.2014.2030

**Published:** 2014-10-17

**Authors:** JAE-SUK CHOI, JOO WAN KIM, HYUNG-RAE CHO, KI-YOUNG KIM, JONG-KWANG LEE, JAE HAK SOHN, SAE-KWANG KU

**Affiliations:** 1 RIS Center, IACF, Silla University, Sasang-gu, Busan 617-736, Republic of Korea; 2 Department of Bio-Food Materials, Silla University, Sasang-gu, Busan 617-736, Republic of Korea; 3 Glucan Corporation, Marine Bio-Industry Development Center, Gijang-gun, Busan 619-912, Republic of Korea; 4 JKnutra, Songpa-gu, Seoul 138-160, Republic of Korea; 5 Department of Anatomy and Histology, College of Oriental Medicine, Daegu Haany University, Gyeongsan, Gyeongsangbuk-do 712-715, Republic of Korea

**Keywords:** fermented rice extract, laxative, constipation, histomorphometry, rat

## Abstract

Constipation is a common problem in males and females. The aim of the present study was to evaluate the laxative effects of fermented rice extract (FRe) on rats with loperamide-induced constipation. FRe (100, 200 and 300 mg/kg) was administered orally once per day for six days following 1 h loperamide treatment. The laxative effects of FRe were compared with those of sodium picosulfate (S. picosulfate). Following the induction of constipation in the rats, a marked decrease was observed in the fecal pellet number and water content discharged over 24 h, the surface mucus thickness in the colonic lumen, intestinal charcoal transit ratio, thickness of the colonic mucosa and the number of mucus-producing cells, while an increase was observed in the number of fecal pellets remaining in the colonic lumen and their mean diameter, as compared with the normal vehicle control rats. These conditions were significantly alleviated following the administration of the three doses of FRe when compared with the loperamide control group. However, the alleviating effects were lower than those of S. picosulfate, with the exception of the intestinal charcoal transit ratio. Similar effects on the intestinal charcoal transit ratio were detected for the three doses of FRe when compared with the S. picosulfate-treated rats. In conclusion, the results indicated that FRe exhibits a laxative effect without causing diarrhea, as compared with sodium picosulfate; thus, FRe may be effective as a complementary medicine in patients suffering from lifestyle-induced constipation.

## Introduction

Constipation is a common health problem with a tendency to cause discomfort and affect patient quality of life (1). This highly prevalent functional gastrointestinal disorder affects 3–15% of the general population (2,3). Constipation may also cause abdominal distension, vomiting, restlessness, gut obstruction and perforation, and may even be associated with aspiration or fatal pulmonary embolism (4). At present, constipation disproportionately affects older adults, with prevalences of 50% among the community-dwelling elderly and 74% in nursing-home residents (5).

Drugs that contain magnesium oxide or sennoside, the main constituent of Senna, are frequently administered for the treatment of constipation due to their powerful purgative/laxative effects. However, these drugs also induce severe diarrhea as a side-effect (1). Furthermore, repeated use of Senna or other anthraquinone-containing drugs may induce melanosis coli, which is a risk factor for colorectal neoplasia (6).

Loperamide-induced delay in colonic transit is accepted as spastic constipation due to the inhibition of stool frequency and increased colonic contractions in humans (7). The drug inhibits intestinal water secretion (8) and colonic peristalsis (9), which extends the fecal evacuation time and delays intestinal luminal transit (10). Thus, loperamide-induced constipation is considered to be a model of spastic constipation (11).

Fermented rice extracts (FRes) have been shown to possess various pharmacological effects, including antiosteoporotic (12), hypoglycemic (13), antitumor (14), neuroprotective (15), antiatherosclerotic (16), antistress and antifatigue effects (17), when compared with non-fermented extracts.

A previous study revealed the growth-stimulating effects of FRe on lactic acid bacteria and *Bifidobacterium* spp. *in vitro* (18). However, whether FRe is effective for treating constipation *in vivo* remains unknown.

In the present study, changes in fecal (number, weight and water content), peristalsis (gastrointestinal transit ratio) and histological (fecal mucus content, colonic mucus-producing cell number and mean colonic mucosa thickness) parameters were observed in rats with loperamide-induced constipation in order to analyze the laxative effects of FRe. The laxative effects of FRe were compared with those of 5 mg/kg sodium picosulfate (S. picosulfate), a cathartic stimulant that requires activation by colonic bacteria (19,20), as a reference drug (21).

## Materials and methods

### Materials

FRe preparations were supplied by Glucan Corporation (Busan, Korea) as brown powders. Briefly, FRe production was performed in three fermentation steps. In the first fermentation step (saccharification), washed, non-glutinous rice (1 kg) was soaked for 6 h, drained for 30 min and steamed for 15 min at 121°C. Following rapid cooling, 10 g malt powder and 4 liters water were added and the mixture was fermented in a 20-liter sterile glass container at 55°C for 12 h. In the second fermentation step, 20 ml *Saccharomyces cerevisiae* (ATCC^®^ 9804^™^; American Type Culture Collection, Manassas, VA, USA) suspension was added to the first fermentate, mixed well and incubated at 30°C for 48 h. Finally, in the third fermentation step, 20 ml lactic acid bacteria*, Weissella paramesenteroides* (KACC 91704; Korean Agricultural Culture Collection, Suwon, Korea), was added to the second fermentate, mixed well and incubated at 30°C for 48 h. This final fermentate was sterilized using an auto-steam sterilizer (VS-1321-80; Vision Scientific Co. Ltd., Daejeon, Korea) at 150°C for 15 min and filtered through a 40-mesh sieve to obtain the final filtrate, which was freeze-dried for two days until a moisture content of ~0.8% was obtained. The freeze-dried FRe was ground in a mill and passed through a 500-mesh sieve. The sieved material was stored at −20°C until required for further use.

S. picosulfate was purchased from Crown Pharm. Co. Ltd. (Seoul, Korea). FRe and S. picosulfate were stored at −20°C and protected from light and moisture.

### Animals

Animal experiments were performed in accordance with the Korean Food and Drug Administration Guidelines for Good Laboratory Practice (notification no. 2000-116, 2009). In total, 58 male Sprague-Dawley rats (age, 8 weeks; Japan SLC, Inc., Hamamatsu, Japan) were used in the experiments following a 12-day acclimatization period. Animals were housed, with three animals per polycarbonate cage, in a temperature- (20–23°C) and humidity- (40–50%) controlled room. The light/dark cycle was 12/12 h and food (Samyang Foods Co., Ltd., Wonju, Korea) and water were supplied *ad libitum*.

Constipation was induced in the animals through oral administration of 3 mg/kg loperamide hydrochloride (Sigma-Aldrich, St. Louis, MO, USA) daily for six days at 1 h prior to test material administration, as described previously (22,23). The control rats were administered saline.

FRe (100, 200 or 300 mg/kg) was dissolved in distilled water (DW) and administered orally 1 h after loperamide administration, daily for six days. S. picosulfate (5 mg/kg body weight) was also dissolved in DW and administered orally as a treatment reference (21). In the vehicle and constipation groups, the control rats were administered DW only instead of treatment.

Rats were selected based on body weight and divided into six groups (six rats/group) at day 1 prior to the initiation of test material administration. Briefly, excluding overweight and underweight rats, the rats were arranged in order of weight, and the heaviest six rats were randomly assigned to each of the six groups. The six next heaviest rats were then randomly assigned to each of the six groups; this division process continued until the 36 rats had been randomly assigned to the six groups. The vehicle control group received DW administration, while the constipation control group received loperamide treatment and DW administration. The S. picosulfate group received loperamide treatment and S. picosulfate administration. Finally, there were three FRe treatment groups that received loperamide treatment followed by FRe at a concentration of 100, 200 or 300 mg/kg. This study was approved by the Ethics Committee of Daegu Haany University (Gyeongsan, Korea)

### Body weight measurements

Changes in body weight were measured once per day from one day prior to the initiation of test material administration (D0) to the termination (D6) of the experiment, using an automatic electronic balance (Precisa Instruments, Dietikon, Switzerland). At termination, all experimental animals fasted overnight with unrestricted access to water in preparation for intestinal charcoal transit ratio measurements. Body weight gain (g) was calculated by subtracting the body weight on the first day of administration (D1) from that at D6.

### Fecal parameter measurements

Excreted fecal pellets of the individual rats over 24 h were collected on day 5 of administration (D5). The total number, wet weight and water content of the fecal pellets were determined. The water content was calculated as follows: Fecal water content (%) = [(fecal wet weight - fecal dry weight)/fecal wet weight] × 100.

### Measurement of intestinal charcoal transit ratio

Assessment of the gastrointestinal propulsion of a charcoal meal was determined according to the method proposed by Sagar *et al* (24), with minor modifications. Animals fasted for 18 h prior to the experiment, but consumed water *ad libitum*. At 10 min following the last drug (FRe/S. picosulfate/DW) administration, the animals were fed 1 ml charcoal meal (3% suspension of activated charcoal in 0.5% aqueous methylcellulose; Sigma-Aldrich). At 30 min after the charcoal meal administration, the animals were sacrificed by cervical dislocation and the total intestine length (pyloric sphincter to cecum) and charcoal meal transit distance were measured. The intestinal charcoal transit ratio was calculated as follows: Charcoal transit ratio (%) = [(total small intestine length - transited distance of charcoal meal)/total small intestine length] × 100.

### Measurement of fecal pellets in the large intestine

With the intestinal charcoal transit ratio measurement, the total number and mean thickness (short axis) of fecal pellets remaining in the colon lumen were measured.

### Histological procedures

Assessments of the colonic mucosa and fecal pellets remaining in the colon lumen were determined according to the method described by Wu *et al* (25), with minor modifications. Briefly, segments of the rat distal colon containing one fecal pellet were isolated by ligatures, removed, and immediately fixed in 10% formaldehyde. The fixed tissue was then embedded, serially cross-sectioned (3–4 μm) and stained with alcian blue stain (pH 2.5) (05500; Sigma-Aldrich). The histological profiles were subsequently interpreted by a histopathologist who was blinded to the group distribution.

The mean thickness of the mucosal layer at the fecal surface (μm/fecal pellets), number of mucus-producing cells (cells/mm^2^ of colonic mucosa) and the colonic mucosa thickness (μm/colon) were measured histomorphometrically using an automated image analyzer (DMI-300; DMI, Daegu, Korea) under a microscope (Eclipse 80i, Nikon Corp., Tokyo, Japan).

### Statistical analysis

All data are expressed as the mean ± standard deviation. Multiple comparison tests were conducted for the different dose groups. Homogeneity of variance was examined using Levene’s test. When Levene’s test indicated no significant deviation from the homogeneity of variance, the obtained data were analyzed by one-way analysis of variance followed by Fisher’s least significant difference multi-comparison test to identify pairs of group comparisons that differed significantly. When Levene’s test indicated significant deviations from the homogeneity of variance, a non-parametric comparison test, the Kruskal-Wallis H-test, was used. When a statistically significant difference was observed in the Kruskal-Wallis H-test, the Mann-Whitney U test was used to identify pairs of group comparisons that differed significantly. Statistical analyses were conducted using SPSS for Windows software (Release 14K; SPSS, Inc., Chicago, IL, USA) and P<0.05 was considered to indicate a statistically significant difference.

## Results

### Effects on body weight

A statistically significant (P<0.01 or 0.05) decrease in body weight was detected in the S. picosulfate group, when compared with the vehicle and loperamide control groups. However, no significant change in body weight was detected when comparing any of the FRe-treated groups with the vehicle and loperamide control groups (Table I).

Body weight gain increased by 7.75% in the loperamide control group compared with the vehicle control group, but decreased by 81.08, 0.90, 1.80 and 6.31% in the S. picosulfate and FRe 100-, 200- and 300-mg/kg-treated groups, respectively, as compared with the loperamide control group.

### Effects on fecal parameters

Statistically significant (P<0.01) decreases in the fecal number and water content collected during 24 h were detected in the loperamide control group when compared with the vehicle control group. By contrast, statistically significant (P<0.01 or 0.05) increases in the fecal number and water content were detected on D5 in the S. picosulfate and all FRe-treated groups when compared with the loperamide control group (Table II).

The total number of fecal pellets collected over 24 h on D5 decreased by 21.97% in the loperamide control group when compared with the vehicle control group, and increased by 67.86, 24.91, 34.29 and 58.11% in the S. picosulfate and FRe 100-, 200- and 300-mg/kg-treated groups, respectively, as compared with the loperamide control group.

The total water content of the fecal pellets collected over 24 h on D5 decreased by 32.29% in the loperamide control group when compared with the vehicle control group, and increased by 182.65, 72.14, 97.73 and 91.23% in the S. picosulfate and FRe 100-, 200- and 300-mg/kg-treated groups, respectively, as compared with the loperamide control group.

### Effects on fecal pellets remaining in the colon lumen

Statistically significant (P<0.01) increases in the fecal number and mean diameter of the pellets remaining in the colon lumen were detected in the loperamide control group when compared with the vehicle control group. By contrast, statistically significant (P<0.01) decreases in the fecal number and mean diameter of the pellets remaining in the colon lumen at sacrifice were detected in the S. picosulfate and all FRe-treated groups when compared with the loperamide control group (Table III).

The total number of fecal pellets remaining in the colon lumen increased by 115.79% in the loperamide control group when compared with the vehicle control group, and decreased by 80.53, 73.21, 87.85 and 78.04% in the S. picosulfate and FRe 100-, 200- and 300-mg/kg-treated groups, respectively, when compared with the loperamide control group.

The mean diameter of the fecal pellets remaining in the colon lumen increased by 30.70% in the loperamide control group when compared with vehicle control group, but decreased by 40.24, 28.40, 36.17 and 31.57% in the S. picosulfate and FRe 100-, 200- and 300-mg/kg-treated groups, respectively, when compared with the loperamide control group.

### Effects on the intestinal charcoal transit ratio

A statistically significant (P<0.01) decrease in the intestinal charcoal transit ratio was detected in the loperamide control group when compared with the vehicle control group. By contrast, statistically significant (P<0.01 or <0.05) increases in the intestinal charcoal transit ratio were detected after six days of continuous oral treatment with S. picosulfate and in all the FRe-treated groups when compared with the vehicle control group (Table IV).

The intestinal charcoal transit ratio decreased by 20.87% in the loperamide control group when compared with the vehicle control group, and increased by 15.10, 16.40, 22.65 and 18.74% in the S. picosulfate and FRe 100-, 200- and 300-mg/kg-treated groups, respectively, as compared with the loperamide control group.

### Effects on histopathology

**Figure 1:**
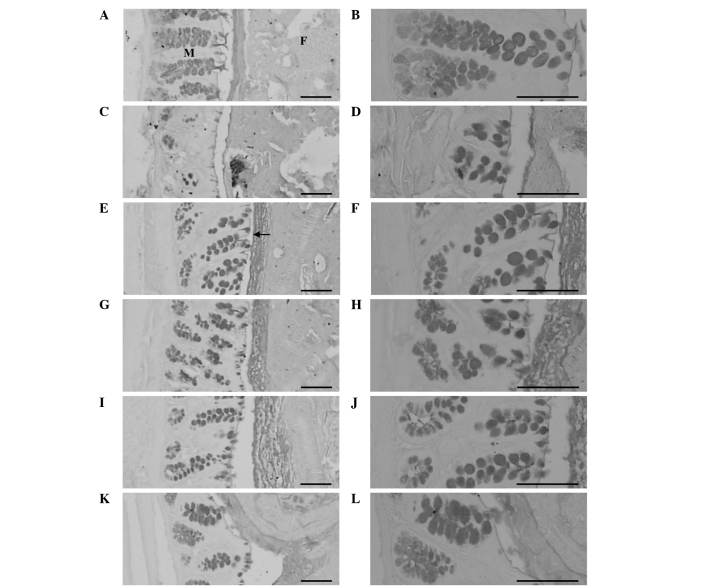
Histological profiles of colons containing fecal pellets from the (A and B) vehicle control, (C and D) loperamide control, (E and F) sodium picosulfate (S. picosulfate), (G and H) fermented rice extract (FRe) 100- , (I and J) 200- and (K and L) 300-mg/kg groups of rats with loperamide-induced constipation. Marked decreases were observed in the surface mucus thickness of the remnant fecal pellets in the colon lumen, the mucosa thickness and the mucus-producing cell number in the loperamide control group when compared with the vehicle control group. By contrast, increases in the surface mucus thickness of the fecal pellets remaining in the colon lumen, the mucosa thickness and the number of mucus-producing cells were detected after six days of continuous oral treatment with S. picosulfate and the three doses of FRe, when compared with the loperamide control group. Oral administration of FRe was performed daily for six days following loperamide treatment. Oral administration of S. picosulfate (5 mg/kg) was performed daily for six days following loperamide treatment. Arrow indicates the surface mucus thickness measurement. Alcian blue staining; Scale bars = 150 μm. M, colonic mucosa; F, fecal pellets.

Statistically significant (P<0.01) decreases in the surface mucus thickness of the fecal pellets remaining in the colon lumen, the mucosa thickness and the number of mucus-producing cells were detected in the loperamide control group when compared with the vehicle control group. By contrast, statistically significant (P<0.01 or <0.05) increases in the surface mucus thickness of the fecal pellets remaining in the colon lumen, the mucosa thickness and the number of mucus-producing cells were detected after six days of continuous oral treatment with S. picosulfate and in all the FRe-treated groups when compared with the loperamide control group (Table V; Fig. 1).

The surface mucus thickness of the fecal pellets remaining in the colon lumen decreased by 73.71% in the loperamide control group when compared with the vehicle control group, and increased by 813.55, 478.17, 677.88 and 506.37% in the S. picosulfate and FRe 100-, 200- and 300-mg/kg-treated groups, respectively, as compared with the loperamide control group.

The number of mucus-producing cells in the colonic mucosa decreased by 69.84% in the loperamide control group when compared with vehicle control group, and increased by 100.10, 42.27, 92.06 and 74.12% in the S. picosulfate and FRe 100-, 200- and 300-mg/kg-treated groups, respectively, as compared with the loperamide control group.

The thickness of the colonic mucosa decreased by 53.68% in the loperamide control group when compared with the vehicle control group, and increased by 110.91, 50.39, 86.92 and 85.38% in the S. picosulfate and FRe 100-, 200- and 300-mg/kg-treated groups, respectively, as compared with the loperamide control group.

## Discussion

Constipation is a common health problem with a tendency to cause discomfort and affect patient quality of life (23). The occurrence of constipation increases with age and may develop into a chronic condition requiring the long-term use of laxatives. Constipation may arise from a variety of causes, including the use of chemical compounds, such as morphine, dietary habits and psychological stress (1).

Various fermentations of rice have increased bioavailabilities and pharmacological activities (12–18). In the present study, the laxative effects of FRe were evaluated based on changes in fecal parameters (numbers, weight and water content), the gastrointestinal transit ratio (motility), fecal mucus content, number of colonic mucus-producing cells and the mean colonic mucosa thickness in rats with loperamide-induced constipation, used as a model of spastic constipation (11). The laxative effects of FRe were compared with those of S. picosulfate, a cathartic stimulant that requires activation by colonic bacteria (19,20), as a reference drug (21).

As a result of loperamide treatment, a marked decrease was detected in the fecal pellet number and water content discharged over 24 h, the surface mucus thickness in the colonic lumen, the intestinal charcoal transit ratio, the thickness of the colonic mucosa and the number of mucus-producing cells. By contrast, an increase was observed in the number of fecal pellets remaining in the colon and their mean diameters in the colonic lumen, as compared with the normal vehicle control. Therefore, the observations indicated a successful model of constipation was established. However, marked increases were observed in the three groups of FRe-treated rats with regard to the fecal pellet number and water content discharged over 24 h, the surface mucus thickness in the colonic lumen, the intestinal charcoal transit ratio, thickness of the colonic mucosa and the number of mucus-producing cells, while decreases were observed in the remaining fecal pellet number and their mean diameters in the colonic lumen, when compared with loperamide control. These changes were less significant compared with those in the S. picosulfate group, with the exception of the intestinal charcoal transit ratio. Similar effects in the intestinal charcoal transit ratio were detected at all three doses of FRe and with S. picosulfate. These results provide direct evidence that compared with S. picosulfate, FRe exhibits a laxative effect without causing diarrhea; thus, may be highly effective as a complementary treatment for humans suffering from lifestyle-induced constipation.

The optimal effective dose of FRe was considered to be ~100 mg/kg since marked dose-dependent effects were detected between 100 and 200 mg/kg, but not between 200 and 300 mg/kg in the present study. FRe did not induce severe diarrhea as a side-effect, possibly since milder and more favorable laxative effects were demonstrated compared with S. picosulfate. In addition, FRe did not influence body weight gain in the present study, whereas S. picosulfate induced a marked reduction in body weight gain due to its powerful purgative/laxative activity.

A marked decrease in fecal discharge was observed during constipation, and the delay of the fecal pellets in the large intestinal lumen induced the over-absorption of water from the fecal pellets. Accordingly, the water content in the discharged fecal pellets was markedly decreased. Changes in the fecal parameters, including the number of discharged fecal pellets and the water content, have been used as an index to detect the effects of various laxative agents (23,25). In the present study, increases in the number of discharged fecal pellets and water content were detected with FRe treatment, and these changes were considered to be direct evidence that FRe exhibits favorable laxative effects. Furthermore, increased numbers of fecal pellets remaining in the colonic lumen and a decrease in the surface mucus content have been detected in individuals with constipation (25,26). The increase in the surface mucus content and decrease in the number of fecal pellets remaining in the colonic lumen following treatment with FRe were considered as direct evidence that FRe has favorable laxative effects.

The transit process of the entire gastrointestinal tract reflects the overall gastrointestinal motor activity. Thus, measuring the gastrointestinal charcoal transit ratio is useful in the diagnosis of constipation (23). A decrease in the gastrointestinal charcoal transit ratio indicates constipation (21,24). In the present study, an increase in the gastrointestinal charcoal transit ratio was observed in the rats treated with FRe, providing indirect evidence that FRe exerts a favorable laxative effect.

Reduced mucus production in the colonic mucosa is directly associated with constipation (26), and marked decreases in the thickness of the colonic mucosal layer and the number of mucus-producing cells have been detected by histopathology in individuals with constipation (27). Thus, in the present study, increases in the number of mucus-producing cells and the thickness of the mucosal layer in the rats following FRe treatment was considered as direct evidence that FRe exhibits a favorable laxative effect.

A number of bifidogenic growth stimulators have been identified, including 2-amino-3-carboxy-1,4-naphthoquinone, 1,4-dihydroxy-2-naphthoic acid (28) and panose (29). Panose has been demonstrated to exert bifidogenic effects and significantly increase the growth of *Bifidobacterium* sp. *in vitro* (29). Furthermore, the ingestion of bifidogenic growth stimulators has been demonstrated to increase the number of defecations in females with constipation (30) and mitigate inflammatory bowel disease (31) via an improvement of the intestinal microflora.

In a previous study (18), a FRe containing 6.7% panose was found to stimulate the growth of lactic acid bacteria, including *Lactobacillus acidophilus*, *Streptococcus thermophilus* and *Bifidobacterium lactis*, when compared with the control group. A 13.8-16 fold increase was observed in growth following incubation for 12 and 18 h. Therefore, FRe as a bifidogenic growth stimulator, was hypothesized to mediate its laxative effects in the current study.

In conclusion, a number of factors, including diet, malnutrition, metabolic or endocrine disorders and side effects of medication, can lead to constipation. The results of the present study indicate that FRe can exert a treatment effect on certain types of constipation induced by the opioid drug loperamide. When compared with S. picosulfate, FRe exhibited laxative effects without causing diarrhea. Thus, FRe may be effective as a complementary treatment for certain types of constipation in humans. The optimal effective dosage of FRe is considered to be ~100 mg/kg since marked dose-dependent effects were detected between 100 and 200 mg/kg in the current study. Further studies are required to elucidate whether FRe causes a healing effect in constipation induced by other factors.

## Figures and Tables

**Table I:** Body weight change following oral treatment with FRe or S. picosulfate in rats with loperamide-induced constipation.

	Body weight (g)			
		
Groups	D1^a^	D5	D6^a^	Difference [D6 - D1]
Controls				
Vehicle	250.50±11.67	294.33±14.38	267.67±12.44	17.17±1.94
Loperamide	248.33±8.41	291.17±9.02	266.83±8.52	18.50±2.43
S. picosulfate	250.33±9.35	277.50±12.36^b^,^c^	253.83±12.27^b^	3.50±3.56^d^,^e^
FRe (mg/kg)				
100	252.17±5.42	292.00±5.97	270.50±7.06	18.33±6.74
200	250.17±9.06	290.83±13.48	268.33±13.02	18.17±6.88
300	247.33±10.27	286.00±10.64	264.67±12.16	17.33±5.65

Values are expressed as the mean ± standard deviation (n=6).

a Overnight-fasted;

b P<0.05, vs. vehicle (Fisher’s least significant difference test);

c P<0.05, vs. loperamide control (Fisher’s least significant difference test);

d P<0.01, vs. vehicle (Mann-Whitney U test);

e P<0.01, vs. loperamide control (Mann-Whitney U test).

Dn, day; FRe, fermented rice extract (daily oral administration for six days following loperamide treatment); S. picosulfate, sodium picosulfate (5 mg/kg oral daily administration for six days following loperamide treatment).

**Table II:** Fecal parameters following oral treatment with FRe or S. picosulfate in rats with loperamide-induced constipation.

	Fecal parameters on D5 (collection for 24 h)			
	
Groups	Pellets (n)	Wet weight (g/24 h/rat)	Dry weight (g/24 h/rat)	Water content (%)
Controls				
Vehicle	59.17±3.19	10.006±0.954	5.855±0.496	41.311±4.106
Loperamide	46.17±6.40^a^	7.673±1.110^b^	5.560±0.270	26.320±10.426^a^
S. picosulfate	77.50±23.45^c^	25.991±5.432^a^,^c^	6.575±1.072	74.394±2,769^a^,^c^
FRe (mg/kg)				
100	57.67±9.18	9.964±1.976	5.392±0.748	45.307±4.113^c^
200	62.00±7.90^c^	13.134±2.721^b^,^c^	6.265±1.153	52.042±1.885^a^,^c^
300	73.00±5.87^a^,^c^	13.651±1.791^a^,^c^	6.725±0.597^b^,^c^	50.332±4.768^a^,^c^

Values are expressed as the mean ± standard deviation (n=6).

a P<0.01 and

b P<0.01, vs. vehicle (Mann-Whitney U test);

c P<0.01 vs. loperamide control (Mann-Whitney U test).

Dn, day; FRe, fermented rice extract (daily oral administration for six days following loperamide treatment); S. picosulfate, sodium picosulfate (5 mg/kg oral daily administration for six days following loperamide treatment).

**Table III:** Fecal pellets in the colon following oral treatment with FRe or S. picosulfate in rats with loperamide-induced constipation.

	Fecal pellets in the colon	
	
Groups	Pellets (n)	Mean thickness (μm)
Controls		
Vehicle	3.17±1.47	4.86±0.59
Loperamide	6.83±0.98^a^	6.35±0.50^d^
S. picosulfate	1.33±1.21^a^,^c^	3.80±0.47^e^,^f^
FRe (mg/kg)		
100	1.83±1.17^b^,^c^	4.55±0.75^f^
200	0.83±0.75^a^,^c^	4.05±0.35^b^,^f^
300	1.50±0.84^b^,^c^	4.35±0.25^f^

Values are expressed as the mean ± standard deviation (n=6).

a P<0.01 and

b P<0.05, vs. vehicle (Fisher’s least significant difference test);

c P<0.01, vs. loperamide control (Fisher’s least significant difference test);

d P<0.01 and

e P<0.05, vs. vehicle (Mann-Whitney U test);

f P<0.01, vs. loperamide control (Mann-Whitney U test).

FRe, fermented rice extract (daily oral administration for six days following loperamide treatment); S. picosulfate, sodium picosulfate (5 mg/kg oral daily administration for six days following loperamide treatment).

**Table IV:** Gastrointestinal charcoal transit ratio following oral treatment with FRe or S. picosulfate in rats with loperamide-induced constipation.

	Gastrointestinal motility (during 30 min)		
	
Groups	Total small intestine length (cm)	Transit distance of charcoal meal (cm)	Gastrointestinal charcoal transit ratio (%)
Controls			
Vehicle	113.17±11.00	78.40±10.66	69.25±6.42
Loperamide	112.07±4.67	61.15±5.61^a^	54.80±7.01^a^
S. picosulfate	111.83±5.45	70.58±9.41^c^	63.07±7.27^c^
FRe (mg/kg)			
100	110.58±7.51	70.42±4.75^c^	63.79±4.07^c^
200	111.75±5.18	74.75±6.12^a^	67.21±8.81^b^
300	112.08±5.15	72.83±6.73^c^	65.07±6.44^c^

Values are expressed as the mean ± standard deviation (n=6). Charcoal transit ratio (%) = [(total small intestine length - transited distance of charcoal meal)/total small intestine length] × 100.

a P<0.01, vs. vehicle (Fisher’s least significant difference test);

b P<0.01 and

c P<0.05, vs. loperamide control (Fisher’s least significant difference test).

FRe, fermented rice extract (daily oral administration for six days following loperamide treatment); S. picosulfate, sodium picosulfate (5 mg/kg oral daily administration for six days following loperamide treatment).

**Table V:** Histomorphometry of colon and remaining fecal pellets following oral treatment with FRe or S. picosulfate in rats with loperamide-induced constipation.

	Histomorphometry (at sacrifice)		
	
Groups	Fecal pellet surface mucus thickness (μm)	Mucus-producing cell numbers (cells/mm^2^)	Colon mucosa thickness (μm)
Controls			
Vehicle	64.71±14.09	643.20±117.17	499.08±102.84
Loperamide	17.27±2.97^d^	194.00±14.73^d^	231.16±42.32^a^
S. picosulfate	157.77±48.75^d^,^f^	388.20±55.56^d^,^f^	487.54±95.24^b^
FRe (mg/kg)			
100	99.85±12.08^d^,^f^	276.00±51.35^d^,^f^	347.64±54.21^a^,^c^
200	134.34±44.04^e^,^f^	372.60±89.34^d^,^f^	432.08±74.44^b^
300	104.72±14.41^d^,^f^	337.80±60.41^d^,^f^	428.53±102.52^b^

Values are expressed as the mean ± standard deviation (n=6).

a P<0.01, vs. vehicle (Fisher’s least significant difference test);

b P<0.01 and

c P<0.05, vs. loperamide control (Fisher’s least significant difference test);

d P<0.01 and

e P<0.05, vs. vehicle control (Mann-Whitney U test);

f P<0.01, vs. loperamide control (Mann-Whitney U test).

FRe, fermented rice extract (daily oral administration for six days following loperamide treatment); S. picosulfate, sodium picosulfate (5 mg/kg oral daily administration for six days following loperamide treatment).
